# Possible explanation of excess events in the search for jets, missing transverse momentum and a *Z* boson in *pp* collisions

**DOI:** 10.1140/epjc/s10052-015-3591-6

**Published:** 2015-08-12

**Authors:** Ulrich Ellwanger

**Affiliations:** LPT, UMR 8627, CNRS, Université de Paris–Sud, 91405 Orsay, France; School of Physics and Astronomy, University of Southampton, Highfield, Southampton, SO17 1BJ UK

## Abstract

We study to which extent SUSY extensions of the Standard Model can describe the excess of events of 3.0 standard deviations observed by ATLAS in the on-*Z* signal region, respecting constraints by CMS on similar signal channels as well as constraints from searches for jets and $E^\mathrm{miss}_\text {T}$. GMSB-like scenarios are typically in conflict with these constraints, and do not reproduce well the shape of the $E^\mathrm{miss}_\text {T}$ distribution of the data. An alternative scenario with two massive neutralinos can improve fits to the total number of events as well as to the $H_\text {T}$ and $E^\mathrm{miss}_\text {T}$ distributions. Such a scenario can be realised within the NMSSM.

## Introduction

After the first run of the LHC at a center of mass (c.m.) energy of mostly 8 TeV, no significant excesses have been observed in searches for physics beyond the standard model [1, 2]. These searches cover a wide range of possible signatures, notably various combinations of jets, missing transverse energy ($E^\mathrm{miss}_\text {T}$), *b*-jets and leptons (electrons or muons).

Same-flavour opposite-sign dileptons can be classified into “off-*Z*” leptons (typically with an invariant mass $m_{ll} < 81$ GeV or $m_{ll} > 101$ GeV), and “on-*Z*” leptons with $81\ \text {GeV} < m_{ll} < 101$ GeV. Often, leptons and in particular on-*Z* dileptons are vetoed in order to suppress Standard Model (SM) backgrounds. On the other hand, some decay cascades of supersymmetric (SUSY) particles could be particularly rich in off-*Z* dileptons (in the presence of light sleptons), or on-*Z* dileptons if *Z* bosons appear particularly frequently in these cascades.

Recently, results of searches for SUSY particles in events with dileptons, jets and $E^\mathrm{miss}_\text {T}$ have been published by the CMS and ATLAS collaborations [3, 4]. The aim was to test scenarios of gluino pair production in which the gluinos $\tilde{g}$ decay via sleptons (leading to off-*Z* dileptons), and scenarios of gauge mediated SUSY breaking (GMSB) or generalised gauge mediation (GGM) where the gluinos undergo 3-body decays into quark pairs and a neutralino $\chi _1^0$. The latter may decay subsequently into a nearly massless gravitino $\tilde{G}$ and a *Z* boson, leading to on-*Z* dileptons. The corresponding gluino decay chain is then $\tilde{g} \rightarrow q+\bar{q}+\chi _1^0 \rightarrow q+\bar{q}+Z+\tilde{G}$. Relevant parameters are the gluino mass $m_{\tilde{g}}$, the neutralino mass $m_{\chi _1^0}$, and the branching fractions of the involved decays.

Whereas no significant excesses were observed by CMS in [3] (up to an excess of 2.6 standard deviations in the dilepton mass window $20\ \text {GeV} < m_{ll} < 70$ GeV), an excess of 3.0 standard deviations was reported by ATLAS in [4] in the on-*Z* signal region: summing electron and muon pairs, 29 events passing the cuts were observed versus $10.6 \pm 3.2$ background events expected. No attempt was made in [4] to explain the excess in terms of a specific model; instead, weaker exclusion limits than expected were shown in the $m_{\tilde{g}}-m_{\chi _1^0}$ plane of GGM models. Various studies of scenarios which could contribute to this excess have recently been published [5–13].

*Z* bosons decay dominantly hadronically. Thus, whenever gluinos are pair produced, in most cases each of the two gluino cascades will produce no dileptons, but two hard jets: either from $q+\bar{q}$ if $m_{\tilde{g}}\gg m_{\chi _1^0} \gtrsim M_Z$, or from hadronic *Z* decays if $m_{\tilde{g}}\gtrsim m_{\chi _1^0} \gg M_Z$ implying a neutralino much heavier than the gravitino, i.e. energetic *Z* bosons. Hence, both scenarios are subject to constraints from “standard” searches for SUSY in events with hard jets and $E^\mathrm{miss}_{\text{ T }}$ [14, 15], even if one considers simplified models where squarks are assumed to be decoupled and gluino pair production is the only process taken into account.

In order to study the impact of these constraints on GMSB-like scenarios, we simulated various configurations of gluino and $\chi _1^0$ masses. Using the latest version 1.2.0 of CheckMATE [16] we found that constraints from [17] (a preliminary version of [14]) on final states with jets and $E^\mathrm{miss}_{\text{ T }}$ are very restrictive, and supersede even the recent CMS constraints from [3] in the $m_{\tilde{g}}-m_{\chi _1^0}$ plane. Exceptions are scenarios with reduced branching fractions for the considered decay chain, without allowing for alternative final states leading to jets and $E^\mathrm{miss}_{\text{ T }}$.

In the present paper we study to which extent a scenario with two heavy neutralinos in the gluino decay cascade can contribute to the ATLAS signal region, circumventing constraints from searches for jets and $E^\mathrm{miss}_{\text{ T }}$. The gluino decay cascade considered subsequently is of the form

1.1 $$\begin{aligned} \tilde{g} \rightarrow q+\bar{q}+\chi _2^0 \rightarrow q+\bar{q}+Z+\chi _1^0 \end{aligned}$$

with

**Fig. 1:**
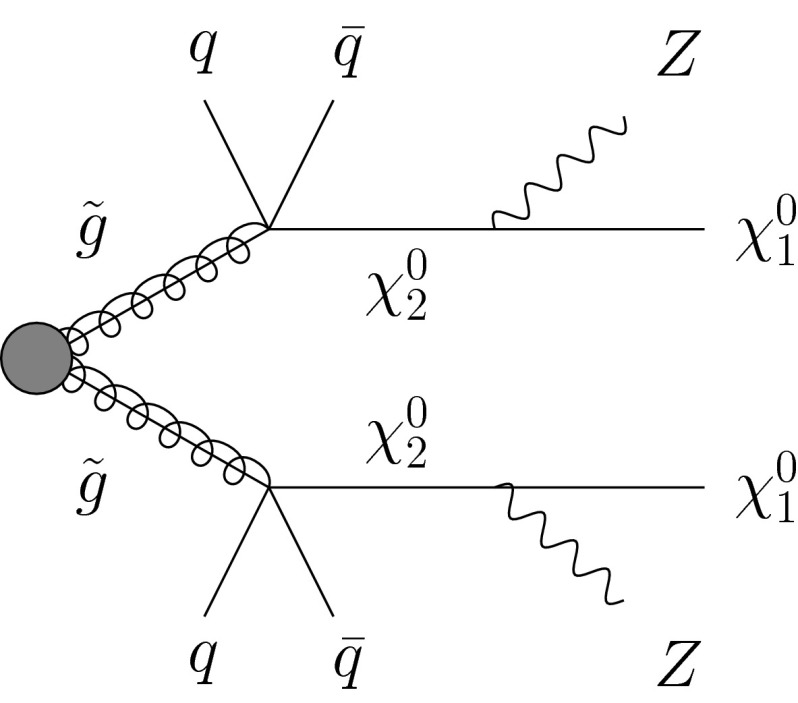
Gluino decay cascades involving two neutralinos $\chi _2^0$ and $\chi _1^0$

1.2 $$\begin{aligned} m_{\chi _2^0} \lesssim m_{\tilde{g}},\qquad m_{\chi _1^0} \sim m_{\chi _2^0} -100\;\text {GeV} \end{aligned}$$

and sketched in Fig. 1.

Now jets from both steps of the gluino decay cascade (including the jets from the *Z* boson) are relatively soft, and constraints from searches for jets and $E^\mathrm{miss}_{\text{ T }}$ are easier to satisfy unless the mass splitting $m_{\tilde{g}}- m_{\chi _2^0}$ is too large. Such a scenario has been considered recently also in [9]. We will compare their results to ours in the conclusions.

In the following we consider first simplified models with 100 % branching fractions for both steps of the gluino decay cascade. We simulated corresponding events, verified which scenarios satisfy the constraints from the CMS [3] and other SUSY searches, and applied the cuts of ATLAS [4]. We will compare the signal rates and various distributions to the data given in [4], and to a GMSB-like scenario (the latter with reduced branching fractions in order to comply with constraints). Constraints from CMS [3] prevent an excess as large as 3.0 standard deviations in the ATLAS signal region, but about 14 signal events on top of the background are possible.

However, the question arises in which SUSY scenario such a neutralino spectrum and, notably, such a dominant gluino decay cascade are possible: What can prevent a dominant $\tilde{g} \rightarrow q+\bar{q}+\chi _1^0$ decay which is favored by phase space? In GMSB the rôle of $\chi _1^0$ is played by the nearly massless gravitino, which has tiny couplings to the MSSM-like sparticles and is not produced unless, due to R-parity conservation, it is the only decay channel. A heavier neutralino $\chi _1^0$ with small couplings to the MSSM-like sparticles, as required in the present scenario, is possible in the NMSSM [18] in the form of the singlino, the fermionic partner of the singlet superfield *S* whose vacuum expectation value generates dynamically a $\mu$-term (a SUSY mass term for the two Higgs doublets in the MSSM) of the order of the SUSY breaking scale. We find that there exist indeed scenarios within the parameter space of the NMSSM for which the gluino decay cascade in Eq. (1.1) is dominant.

In the next section we describe details of the simulation and cuts. Results for simplified models and the description of a NMSSM scenario are given in Sect. 3. We conclude in Sect. 4.

## Simulations and cuts

We have simulated events at the LHC at 8 TeV using MadGraph/MadEvent [19] which includes Pythia 6.4 [20] for showering and hadronisation. The emission of one additional hard jet was allowed in the simulation in order to obtain realistic distributions for kinematical variables. The production cross sections were obtained by Prospino at NLO [21, 22].

First, the output was given to CheckMATE version 1.2.0 [16] which includes the detector simulation DELPHES [23] and compares the signal rates to constraints from various search channels of ATLAS and CMS. All searches present in CheckMATE version 1.2.0 have been verified; the most relevant ones (with the largest ratio for the event yield to $S^{95}_\text {obs}$ where $S^{95}_\text {obs}$ is the observed 95 % CL upper bound) are obtained from the ATLAS search for jets and $E^\mathrm{miss}_{\text{ T }}$ in [17].

Second, the Pythia output was given directly to DELPHES and analysed according to the object identification and selection criteria given in [3, 4], respectively, and finally the corresponding cuts were applied.

For the ATLAS on-*Z* searches [4] these were as follows: $E^\mathrm{miss}_\text {T} > 225$ GeV; $\ge 2$ jets with $p_\text {T} > 35$ GeV; two same-flavour opposite-sign leptons with $p_\text {T} > 25$ GeV for the leading, $p_\text {T} > 10$ GeV for the sub-leading lepton; $H_\text {T} > 600$ GeV where $H_\text {T} = p_\text {T}^\text {lepton,1} + p_\text {T}^\text {lepton,2} + \sum _i p_\text {T}^\text {jet,i}$ (including jets with $p_\text {T} > 35$ GeV); and finally $81\ \text {GeV} < m_{ll} < 101$ GeV. 29 events passing the cuts were observed, whereas $10.6~\pm ~3.2$ background events were expected.

For the events passing the cuts, distributions of $m_{ll}$, $E^\mathrm{miss}_\text {T}$, $H_\text {T}$ and the jet multiplicity $N_{\text {jets}}$ were shown in [4] separately for the electron and muon channels. These distributions were compared with those expected from two GGM benchmark points with gluino masses and neutralino masses of $(m_{\tilde{g}},m_{\chi _1^0})= (700,200)$  GeV, (900, 600)  GeV, respectively. We found, however, that both points violate constraints from [17] on final states with jets and $E^\mathrm{miss}_\text {T}$.

We compared the expected properties of the two GGM benchmark points in [4] to the results of our simulation and found that they agree within $\sim$30 %. We conclude that the results of our simulations deviate by a systematic error of up to $\sim$30 % from the more realistic (detector-) simulation of the experimental collaboration. We can expect that this systematic error cancels to a large extent when comparing the properties of different simulated scenarios, but should be taken into account when comparing to the actual data from [4]. Since it is of the same order (actually somewhat larger) than the difference in the acceptances of dielectrons and dimuons in [4], we found it reasonable to consider the sum of the data of dielectron and dimuon events not only for the signal rate, but also for the kinematical distributions and the expected SM background in order to obtain a larger statistics.

In the CMS on-*Z* searches [3], no cuts on $H_\text {T}$ were applied. Signal jets were required to have $p_\text {T} > 40$ GeV. Six $E^\mathrm{miss}_{\text{ T }}$- and $N_{\text {jets}}$-dependent on-*Z* signal regions were defined: $E^\mathrm{miss}_{\text{ T }}=100-200,\ 200-300,\ >$300 GeV and $N_{\text {jets}} \ge 2,\ \ge$3, respectively. Finally the CMS and ATLAS analyses differ slightly in the jet algorithms and in the lepton acceptances. Comparing the signal rates obtained by our simulations of the two GMSB-like benchmark points to the simulations in [3] we found again that they agree within $\sim$30 %.

As already stated above, no significant excesses were observed in the on-*Z* signal regions by CMS. Hence the event yields in the six on-*Z* signal regions lead to constraints on any scenarios which attempt to explain the ATLAS excess. In the next section we discuss by means of benchmark points to which extent the ATLAS excess can be matched in consideration of these constraints, as well as constraints from [17] on final states with jets and $E^\mathrm{miss}_{\text{ T }}$.

## Results

First we considered GMSB-like simplified models with a branching fraction of 100 % for the $\tilde{g} \rightarrow q+\bar{q}+\chi _1^0 \rightarrow q+\bar{q}+Z+\tilde{G}$ decay chain. Then, however, constraints from the search for jets and $E^\mathrm{miss}_{\text{ T }}$ in [17] as tested by CheckMATE [16] require $m_{\tilde{g}} \gtrsim 1050$ GeV for small $m_{\chi _1^0} \sim 150$ GeV, and larger gluino masses for larger $m_{\chi _1^0}$. Accordingly contributions to the ATLAS signal region cannot exceed $\sim$5 events. Moreover the distribution of $E^\mathrm{miss}_{\text{ T }}$ and $H_\text {T}$ peak towards large values (most events have $H_\text {T}>1500$ GeV) in sharp contrast to the data in [4].

In realistic models, the branching fractions for the steps of the above gluino decay chain can well be below 100 %. Below we will consider a GMSB-like benchmark point “GMSB” with $(m_{\tilde{g}},m_{\chi _1^0})= (800,600)$ GeV and a branching fraction of 10 % for the above decay chain; such a small branching fraction makes it compatible with the CMS constraints. (A heavy $\chi _1^0$ was chosen in order to shift the peak of the $H_\text {T}$ distribution towards lower values.) For the remaining 90 % of the gluino decays one has to expect that, depending on the complete spectrum and branching fractions, they contribute to the signal regions in the search for jets and $E^\mathrm{miss}_{\text{ T }}$ in [17]. One can make the somewhat optimistic assumption that these contributions do not exceed 50 % of the contributions of the $\tilde{g} \rightarrow q+\bar{q}+\chi _1^0 \rightarrow q+\bar{q}+Z+\tilde{G}$ decay chain. Then this point remains within the constraints from [17], but contributes about 10 events to the ATLAS on-Z signal region.

Next we consider simplified models with two heavy neutralinos whose decay chain is depicted in Fig. 1. Assuming a branching fraction of 100 % for this decay chain, gluinos can be as light as 800 GeV without conflict with constraints from the search for jets and $E^\mathrm{miss}_{\text{ T }}$ in [17]—under the condition, however, that $m_{\chi _2^0}$ and $\ m_{\chi _1^0}$ are relatively large such that all jets remain relatively soft. We studied two benchmark points P1 and P2 with $(m_{\tilde{g}},m_{\chi _2^0},m_{\chi _1^0})= (800,790,690)$ GeV and $(m_{\tilde{g}},m_{\chi _2^0},m_{\chi _1^0})= (800,600,500)$ GeV, respectively. Such scenarios belong to the few exceptions allowing for gluinos with a mass below 1 TeV, see the study in [24].

For P1 with $m_{\tilde{g}} - m_{\chi _2^0} = 10$ GeV the jets from the first step $\tilde{g} \rightarrow q+\bar{q}+\chi _2^0$ of the decay cascade are very soft, as are the jets from *Z* decays from the second step $\chi _2^0 \rightarrow Z+\chi _1^0$. Practically all energy of a single gluino decay cascade goes into $E^\mathrm{miss}_{\text{ T }}$. However, for typical kinematical configurations the momenta of $\chi _1^0$ tend to be back-to-back in the transverse plane, leading to a reduction of $E^\mathrm{miss}_{\text{ T }}$ of the complete event. Only for relatively rare kinematical configurations (and/or extra jets from initial state radiation as included in our simulation), $E^\mathrm{miss}_{\text{ T }}$ of the complete event can assume large values. For P2 with $m_{\tilde{g}}-m_{\chi _2^0} = 200$ GeV the jets from the first step $\tilde{g} \rightarrow q+\bar{q}+\chi _2^0$ of the decay cascade are harder, leading to less $E^\mathrm{miss}_{\text{ T }}$. One aim is to study the impact of this difference on the distributions of kinematical variables.

For all benchmark points we assumed practically decoupled squarks with masses of 3 TeV; then the gluino pair production cross section from prospino at NLO is 128 fb. (Since stops and sbottoms are assumed to have masses of 3 TeV as well their pair production does not contribute to the signal.) We deliberately chose identical gluino masses for all points in order to maintain a common production cross section; therefore all differences in contributions to signal regions and kinematical distributions originate from the neutralino sector. The masses of the latter are recalled in Table 1 below.

**Table 1:** Sparticle masses of the benchmark points GMSB, P1 and P2 (in GeV), event yields including 30 % systematic errors from the simulation of the benchmark points GMSB, P1 and P2 in the six signal regions of the CMS on-*Z* searches in [3], and in the most constraining signal regions CT, EM and ET of the ATLAS search [17]. The ranges of $E^\mathrm{miss}_{\text{ T }}$ for the six CMS signal regions are given in GeV. The last line indicates the contributions to the ATLAS on-*Z* signal region

		GMSB	P1	P2
Gluino/neutralino masses				
$m_{\tilde{g}}$		800	800	800
$m_{\chi _1^0}$ (GMSB), $m_{\chi _2^0}$ (P1, P2)		600	790	600
$m_{\tilde{G}}$ (GMSB), $m_{\chi _1^0}$ (P1, P2)		0	690	500
Constraining signal regions	$S^{95}_\text {obs}$			
CMS, $N_{\text{ j }ets} \ge 2$, $100<E^\mathrm{miss}_{\text{ T }}< 200$	207	2.0 $\pm$ 0.6	19.4 $\pm$ 5.8	55.7 $\pm$ 16.7
CMS, $N_{\text{ j }ets} \ge 2$, $200<E^\mathrm{miss}_{\text{ T }}< 300$	20	2.6 $\pm$ 0.78	8.1 $\pm$ 2.4	23.7 $\pm$ 7.1
CMS, $N_{\text{ j }ets} \ge 2$, $300<E^\mathrm{miss}_{\text{ T }}$	7.6	7.0 $\pm$ 2.1	6.1 $\pm$ 1.8	7.0 $\pm$ 2.1
CMS, $N_{\text{ j }ets} \ge 3$, $100<E^\mathrm{miss}_{\text{ T }}< 200$	89	1.9 $\pm$ 0.57	8.5 $\pm$ 2.6	48.4 $\pm$ 14.5
CMS, $N_{\text{ j }ets} \ge 3$, $200<E^\mathrm{miss}_{\text{ T }}< 300$	16.1	2.4 $\pm$ 0.72	4.7 $\pm$ 1.4	21.1 $\pm$ 6.3
CMS, $N_{\text{ j }ets} \ge 3$, $300<E^\mathrm{miss}_{\text{ T }}$	8	6.4 $\pm$ 1.9	3.8 $\pm$ 1.1	6.7 $\pm$ 2.0
ATLAS, CT	2.4	0.73 $\pm$ 0.22	0.79 $\pm$ 0.24	1.31 $\pm$ 0.39
ATLAS, EM	28.6	21.7 $\pm$ 6.5	1.32 $\pm$ 0.39	15.8 $\pm$ 4.7
ATLAS, ET	8.3	7.8 $\pm$ 2.3	0.70 $\pm$ 0.21	4.13 $\pm$ 1.24
ATLAS on-*Z* SR (obs. excess 18.4)		9.8 $\pm$ 2.9	5.6 $\pm$ 1.7	13.6 $\pm$ 4.1

In addition we indicate in the Table 1 in how far the benchmark points GMSB, P1 and P2 satisfy constraints from the six signal regions of the CMS on-*Z* searches in [3] (including 30 % systematic errors from the simulation). The 95 % CL upper limits for the six signal regions of the CMS on-*Z* searches had already been obtained in [9]. We find that the central values of event yields of the benchmark points are below these 95 % CL upper limits with the exception of P2 in the bins $N_\mathrm{jets} \ge 2$, $200<E^\mathrm{miss}_{\text{ T }}< 300$ and $N_\mathrm{jets} \ge 3$, $200<E^\mathrm{miss}_{\text{ T }}< 300$. However, taking the systematic errors from the simulation into account, the $CL_s=CL_{s+b}/CL_b$ values for P2 in these bins are 0.11 and 0.09, respectively, i.e. well above the 95 % CL exclusion limit of 0.05.

Out of the 10 signal regions in the ATLAS search [17] for jets and $E^\mathrm{miss}_{\text{ T }}$ we show the event yields for the signal regions CT, EM and ET which give the largest ratio event yield/$S^{95}_\text {obs}$ for the points P1, P2 and GMSB, respectively. All these signal regions require $E^\mathrm{miss}_{\text{ T }} > 160$ GeV, $p_T > 130$ GeV for the leading jet, and $p_T > 60$ GeV for 3 additional jets (CT), $p_T > 60$ GeV for 5 additional jets (EM and ET). EM and ET differ by $m_\text {eff}(\mathrm{incl.}) > 1200/1500$ GeV, respectively (see [17] for more details).

We recall that the event yields for the point GMSB assume only a branching fraction of 10 % into the considered gluino decay chain. In Table 1, only the contributions from the simulated decay chain are shown. Within the systematic error bars, 50 % more events from other gluino decays are allowed to contribute to the signal regions in the ATLAS search [17] for jets and $E^\mathrm{miss}_{\text{ T }}$ in order to saturate the bound from the signal region ET.

Finally we compare the contributions of the benchmark points GMSB, P1 and P2 to the ATLAS on-*Z* signal region, summing dielectrons and dimuons, in the last line of Table 1.

We see that a price has to be paid for the very compressed gluino $-\chi _2^0-\chi _1^0$ spectrum in P1: Due to the softness of the jets, not enough jets satisfy the cut $N_{\text {jets}} \ge 2$. The GMSB point seems to do quite well, despite its gluino branching fraction being reduced by a factor $\sim$1/10. The best fit is given by P2 with its less compressed gluino $-\chi _2^0-\chi _1^0$ spectrum.

Next we consider the distributions of kinematical variables. As stated above we combine the ATLAS dielectron and dimuon data (despite the different acceptances) in order to enhance the visibility of possible trends. We only show the (dominant) statistical error of the data; we are not in a position to combine the partially correlated systematic errors. In the figures below we show the data with the expected SM background contribution subtracted, with the aim to expose possible desirable features of signal contributions (see [4] for the error attributed to the expected background).

**Fig. 2:**
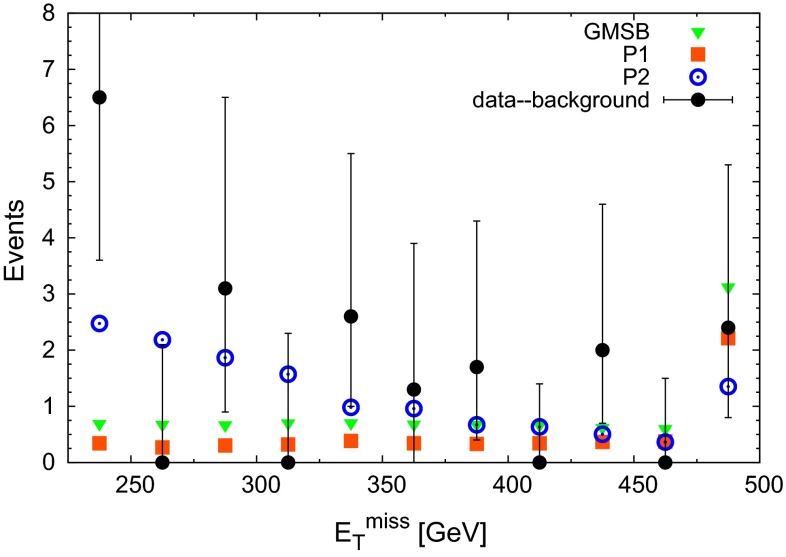
Comparison of $E^\mathrm{miss}_\text {T}$ from the data in [4] (with the expected background subtracted) to the benchmark points GMSB, P1 and P2 defined in the text. *Error bars* on the data are statistical only. The rightmost bin includes the overflow

We start with $E^\mathrm{miss}_\text {T}$ in Fig. 2 where we compare the data with the expected background subtracted to the GMSB scenario and with the two heavy-neutralino benchmark points P1 and P2. We simulated 500.000 events for each scenario. Each expected event for the LHC run I as shown in Fig. 2 corresponds to 10 simulated events, which allows to estimate the statistical errors. These are smaller than the estimated systematic errors from our simulation, and much smaller than the statistical error of the data.

The measured event numbers seem to decrease continuously with $E^\mathrm{miss}_\text {T}$ (within the error bars, and note that the rightmost bin includes the overflow), whereas the $E^\mathrm{miss}_\text {T}$ distributions of the GMSB and P1 points are nearly flat: In these scenarios, nearly all energy is transformed into missing energy which prefers accordingly large values of $E^\mathrm{miss}_\text {T}$. (Note that $E^\mathrm{miss}_{\text{ T }}$ is shown after the application of all cuts, notably on $H_T > 600$ GeV. For P1 with its compressed spectrum this cut selects atypical kinematical configurations with particularly large $E^\mathrm{miss}_{\text{ T }}$.)

For a quantitative comparison we compute the reduced $\chi ^2$ statistic

3.1 $$\begin{aligned} \chi ^2_\text {red} = \frac{1}{N_\text {bins}-1} \sum _{i=1}^{N_\text {bins}}\frac{(N_{d-b}(i)-N_S(i))^2}{\sigma ^2(i)} \end{aligned}$$

for each benchmark point, where $N_{d-b}(i)$ is the data with the expected background subtracted (as shown in Fig. 2). $\sigma (i)$ combines the statistical error of the data shown in Fig. 2 and the systematic error of 30 % of our simulation (with respect to which the systematic error of the background is negligible).

We obtained $\chi ^2_\text {red} = 0.69$ for GMSB, $\chi ^2_\text {red} = 0.85$ for P1 and $\chi ^2_\text {red} = 0.61$ for P2. Hence the scenario P2 with its larger splitting between the gluino and the $\chi _2^0$ masses describes best the shape of the $E^\mathrm{miss}_\text {T}$ distribution. Of course, the scenario P2 profits also from its larger total event rate.

**Fig. 3:**
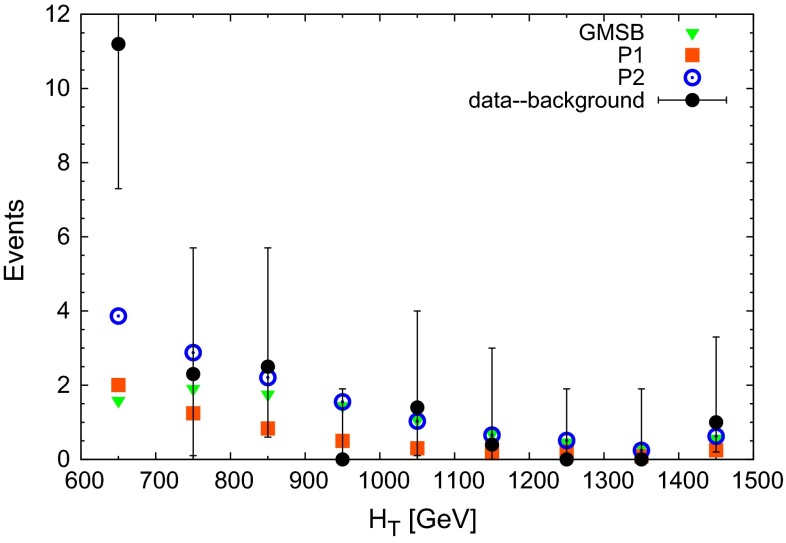
Comparison of $H_\text {T}$ from the data in [4] (with the expected background subtracted) to the benchmark points GMSB, P1 and P2 defined in the text. *Error bars* on the data are statistical only. The rightmost bin includes the overflow

In Fig. 3 we compare the data on $H_\text {T}$ (with the expected background subtracted) with the GMSB scenario and the benchmark points P1 and P2. Since $H_\text {T}$ represents most of the visible transverse energy, the point P1 with its compressed spectrum peaks at low values of $H_\text {T}$. This coincides with the trend of the data, but the total signal rate (limited by constraints from CMS) is small, as indicated in Table 1.

For the reduced $\chi ^2$ statistic we find $\chi ^2_\text {red} = 0.54$ for GMSB, $\chi ^2_\text {red} = 0.69$ for P1 and $\chi ^2_\text {red} = 0.36$ for P2. Again, the benchmark point P2 provides the best agreement with the shape of the distribution despite its somewhat less compressed spectrum.

**Fig. 4:**
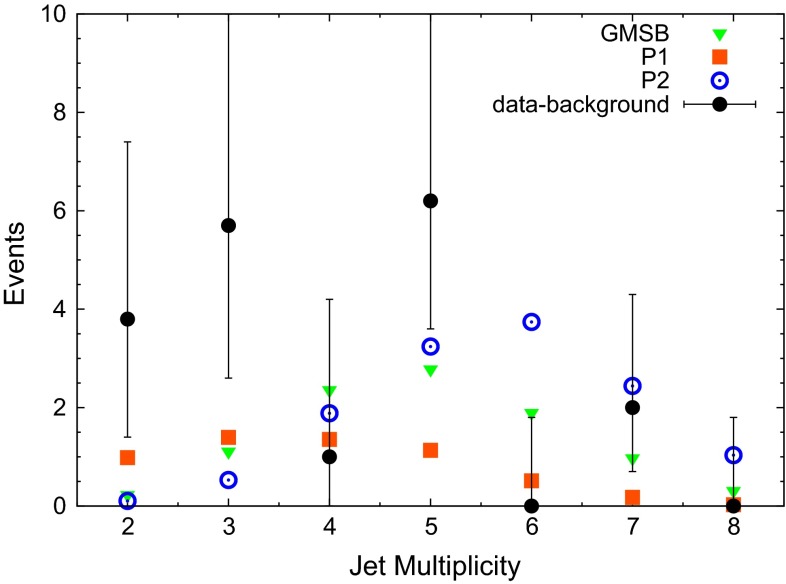
Comparison of the jet multiplicity from the data in [4] (with the expected background subtracted) to the benchmark points GMSB, P1 and P2 defined in the text. *Error bars* on the data are statistical only

Finally we turn to the distribution of the jet multiplicity in Fig. 4. The trend of the data towards low jet multiplicities is reproduced only by P1 with its excessively low signal rate. The jet multiplicity of simulations is sensitive, amongst others, to the matching between soft and hard QCD radiation, accordingly this quantity has to be considered with some reserve.

For the reduced $\chi ^2$ statistic we find $\chi ^2_\text {red} = 1.03$ for GMSB, $\chi ^2_\text {red} = 1.08$ for P1 and $\chi ^2_\text {red} = 1.57$ for P2. In this case the trend of the data is not well reproduced by the point P2. But since the scenario P2 provides the best fit to the ATLAS signal rate and the $E^\mathrm{miss}_\text {T}$ and $H_\text {T}$ distributions, it would be interesting to know about SUSY extensions of the Standard Model which share the features of this simplified model. As discussed in the Sect. 1, this is possible within the NMSSM.

Using the spectrum generator NMSSMTools [25, 26] with decay branching fractions computed by NMSDECAY [27] (based on HDECAY [28]) we found that the following region of the parameter space of the $Z_3$-invariant NMSSM shares the following properties with the point P2:

- Heavy (decoupled) squarks in order to satisfy constraints from searches for events with jets and $E^\mathrm{miss}_\text {T}$ in the presence of a gluino with a mass of 800 GeV.
- A bino-like neutralino $\chi _2^0$ with a mass of 600 GeV, but winos and higgsinos slightly heavier than the gluino. (The running gaugino masses do not satisfy SU(5)-like relations at the GUT scale.)
- A singlet-like neutralino $\chi _1^0$ with a mass of 500 GeV. Then the branching fraction for the decay $\chi _2^0\rightarrow \chi _1^0 +Z$ is 100 %.
- The loop-induced gluino two-body decay $\tilde{g} \rightarrow g + \chi _1^0$ should be suppressed, since it would not contribute to the signal. It is induced by the higgsino component of $\chi _1^0$, and can be of similar order of the desired gluino three-body decay $\tilde{g} \rightarrow q + \bar{q} + \chi _2^0$. The singlino-higgsino mixing is proportional to the NMSSM-specific Yukawa coupling $\lambda$ [18], and $\lambda$ should not exceed $\sim 0.3$. (The loop-induced gluino two-body decay $\tilde{g} \rightarrow g + \chi _2^0$ leads to similar signals as $\tilde{g} \rightarrow q + \bar{q} + \chi _2^0$, but it can be expected that it would improve the jet multiplicity distribution shifting it towards smaller values.)

The remaining parameters can be chosen to obtain a Standard Model-like Higgs boson with a mass of $\sim$125 GeV. We have checked that a corresponding point in the parameter space with all squark masses of 2.5 TeV (leading to a gluino pair production cross section of $\sim$150 fb in order to compensate for a gluino BR into $q + \bar{q} + \chi _2^0$ slightly below 100 %), $\tan \beta =3.75$, $\mu _{\text {eff}}=800$ GeV, $\lambda \sim 0.28$, $\kappa \sim 0.087$, $A_{\lambda } \sim 2.7$ TeV and $A_{\kappa } \sim -50$ GeV (see [18] for the definitions of the latter parameters) has the properties of P2 and would not be distinguishable from P2 regarding the different observables shown above in Figs. 2, 3, 4.

## Summary and conclusions

We studied to which extent SUSY extensions of the SM can describe the excess of events observed by ATLAS in the on-*Z* signal region, respecting constraints by CMS on similar signal channels as well as constraints from searches for jets and $E^\mathrm{miss}_\text {T}$. For viable scenarios we compared the distribution of kinematical variables to the data, combining dielectron and dimuon events.

Due to hadronic *Z*-decays, GMSB-like scenarios are typically in conflict with constraints from searches for jets and $E^\mathrm{miss}_\text {T}$. Assuming a 100 % branching fraction for the gluino decay cascade including the $\chi _1^0 \rightarrow \tilde{G} + Z$ decay, these scenarios become viable only if the gluino has a mass above $\sim$1.05 TeV implying a small contribution ($<$5 events) to the ATLAS signal region. Reducing the branching fraction (including the $\chi _1^0 \rightarrow \tilde{G} + Z$ decay) to $\sim$10 %, lighter gluinos with $m_{\tilde{g}}\sim 800$ GeV may contribute significantly to the signal region, remaining within the 95 % CL limit of CMS. However, the $H_\text {T}$ and notably the $E^\mathrm{miss}_\text {T}$ distributions do not coincide well with the trends of the data. Therefore we studied alternative scenarios with two massive neutralinos $\chi _2^0$, $\chi _1^0$. In order to compare the impact of different neutralino spectra to GMSB-like scenarios and among themselves for fixed gluino pair production cross sections and gluino masses, we fixed the latter also to 800 GeV.

A very compressed $\tilde{g}-\chi _2^0-\chi _1^0$ spectrum reproduces somewhat better the trend of the $H_\text {T}$ distribution, but does not improve the shape of the $E^\mathrm{miss}_\text {T}$ distribution. In particular, the contribution to the ATLAS signal region cannot be enhanced significantly while remaining within the 95 % CL limit of CMS.

A less compressed $\tilde{g}-\chi _2^0-\chi _1^0$ spectrum provides the best fit to the total number of events in the ATLAS in the on-*Z* signal region, as well as to the $H_\text {T}$ and $E^\mathrm{miss}_\text {T}$ distributions; only the jet multiplicity is still not well reproduced. (Larger $\tilde{g}-\chi _2^0$ mass splittings as assumed here become again sensitive to constraints from searches for jets and $E^\mathrm{miss}_\text {T}$.) We found that such a scenario can be realised within the NMSSM.

A somewhat different approach has recently been persued in [9], where the space of the two lightest neutralino masses within the NMSSM was scanned systematically in order to maximise the contribution to the ATLAS on-*Z* signal region respecting existing constraints. The shapes of the kinematical variables have not been studied, however. Still, their main results coincide with ours: Whereas compressed spectra make it easier to satisfy constraints from other SUSY searches, the contributions to the ATLAS on-*Z* signal region are suppressed as well. For gluino masses below $\sim$800 GeV, only a small corner in the plane of the two lightest neutralino masses survives the 95 % CL limits of CMS. Within this corner (for $\tilde{g}$, $\chi _2^0$, $\chi _1^0$ masses of 650, 565 and 465 GeV, respectively) the authors found a maximal contribution of about 11 events to the ATLAS on-*Z* signal region.

Moreover, the authors of [9] considered constraints from signal regions in [14] (2jW and 4jW) which are not implemented in the CheckMATE version 1.2.0 [16] used here. The authors applied these constraints to our benchmark point P2 and obtained a ratio for the event yield/$S^{95}_\text {obs}$ of 1.19, i.e. about 20 % too large, but within the systematic errors from the simulation. A similar excess holds for this point actually also for two CMS signal regions considered in Table 1. We recall that identical gluino masses of 800 GeV were chosen for all points to simplify comparisons. A slightly heavier gluino mass of $\sim$825 GeV would reduce the gluino pair production cross section by $\sim$20 %, but with little changes in the decay kinematics if all mass splittings remain the same. Then this modified point would pass all constraints without the help of systematic error bars, but its contribution to the ATLAS on-*Z* signal region would drop to $\sim$11 events. This number coincides with the maximum found in [9] for the slightly different point above.

Clearly, if the excess observed by ATLAS indicates the presence of particles beyond the standard model, it should become more visible in both ATLAS and CMS experiments at the run II of the LHC. But since it is present in the ATLAS analysis of the available data from run I we found it appropriate to discuss possible interpretations.

Within the class of models considered here, fits to the event numbers and shapes of the ATLAS on-Z can be improved with respect to the GMSB scenarios considered in [4]. However, perfect fits would lead to unacceptable tensions with constraints from other searches.
